# Cross-Crop Transferability of Machine Learning Models for Early Stem Rust Detection in Wheat and Barley Using Hyperspectral Imaging

**DOI:** 10.3390/plants14213265

**Published:** 2025-10-25

**Authors:** Anton Terentev, Daria Kuznetsova, Alexander Fedotov, Olga Baranova, Danila Eremenko

**Affiliations:** 1 All-Russian Institute of Plant Protection, 196608 Saint Petersburg, Russia; afedotov@spbstu.ru (A.F.);; 2 Institute of Cybersecurity and Computer Science, Peter the Great St. Petersburg Polytechnic University, 195251 Saint Petersburg, Russia; kuznetsova_dv@spbstu.ru

**Keywords:** hyperspectral imaging, early plant disease detection, machine learning, hyperspectral data processing, zero-shot cross-domain validation, remote sensing, wheat (*Triticum aestivum* L.), barley (*Hordeum vulgare* L.), stem rust (*Puccinia graminis* f. sp. *tritici*), cross-crop transferability

## Abstract

Early plant disease detection is crucial for sustainable crop production and food security. Stem rust, caused by *Puccinia graminis* f. sp. *tritici*, poses a major threat to wheat and barley. This study evaluates the feasibility of using hyperspectral imaging and machine learning for early detection of stem rust and examines the cross-crop transferability of diagnostic models. Hyperspectral datasets of wheat (*Triticum aestivum* L.) and barley (*Hordeum vulgare* L.) were collected under controlled conditions, before visible symptoms appeared. Multi-stage preprocessing, including spectral normalization and standardization, was applied to enhance data quality. Feature engineering focused on spectral curve morphology using first-order derivatives, categorical transformations, and extrema-based descriptors. Models based on Support Vector Machines, Logistic Regression, and Light Gradient Boosting Machine were optimized through Bayesian search. The best-performing feature set achieved F1-scores up to 0.962 on wheat and 0.94 on barley. Cross-crop transferability was evaluated using zero-shot cross-domain validation. High model transferability was confirmed, with F1 > 0.94 and minimal false negatives (<2%), indicating the universality of spectral patterns of stem rust. Experiments were conducted under controlled laboratory conditions; therefore, direct field transferability may be limited. These findings demonstrate that hyperspectral imaging with robust preprocessing and feature engineering enables early diagnostics of rust diseases in cereal crops.

## 1. Introduction

Wheat (*Triticum aestivum* L.) is one of the most important cereal crops in world agriculture, occupying a leading position in terms of production and consumption worldwide [1]. It is a key food crop, providing about 20% of the world’s calorie and protein consumption [2]. Barley (*Hordeum vulgare* L.) is one of the oldest and most widespread cereal crops globally cultivated [3]. It occupies a significant place in global agricultural production, second in production volumes only to wheat, corn, and rice [4]. About 70% of barley production is used as animal feed, while the remaining ~30% is used in brewing, the food industry, and for direct human consumption [5].

Wheat and barley diseases pose a serious threat to yield stability and grain quality, causing significant economic losses in global agriculture [6,7]. Pathogen infections lead to decreased photosynthetic activity, disruption of ear formation, deterioration of technological properties of grain, and, consequently, reduced food and feed value [4]. Among the many pathogens, a special place is occupied by rust fungi of the genus *Puccinia*, which affect both crops. These diseases are highly destructive, capable of rapid long-distance spread, and may cause epiphytotics, making them key targets of phytosanitary monitoring [8,9].

The causative agent of stem rust, the biotrophic basidiomycete fungus *Puccinia graminis* f. sp. *tritici*, is one of the most harmful wheat pathogens. Yield losses during epiphytotic development on susceptible varieties can reach 80–100%. The pathogen also infects barley and causes yield losses across production regions worldwide [10,11]. The fungus attacks stems, leaves, and glumes of the plant. Like all cereal rust diseases, stem rust produces very characteristic visible symptoms: stems covered with reddish-brown or rusty pustules, ruptures in the plant epidermis filled with powdery unicellular urediniospores. Pustules disrupt transpiration regulation, leading to reduced yield and grain quality, including lower starch, mono- and disaccharides, total and protein nitrogen, and increased non-protein nitrogen. Optimal conditions for the development of *P. graminis* include air temperatures of +18 to +28 °C and free moisturepresence, so dew or evening rains favor the disease. Under favorable conditions, new pustules appear within one week of re-infection [12,13].

The development of rust diseases includes a latent period, i.e., an asymptomatic phase during which the pathogen develops actively inside plant tissues without visible signs of infection. The duration depends on host variety and environmental conditions; for stem rust, it ranges from three to nine days [14,15]. Studies of stem rust have allowed models linking latent period duration to temperature [16]. During this stage, phytosanitary monitoring using visual methods is difficult or impossible, increasing the risk of delayed detection and large-scale spread [17,18].

Phytosanitary monitoring and diagnostics are key components of modern integrated crop protection systems, supporting infection forecasting and disease management. Traditional methods—visual assessment, microscopy of morphological features, and molecular, serological, and microbiological approaches—do not always meet the requirements of modern agriculture [17,18]. Over the past two decades, new technologies have emerged, including remote sensing, digital data processing, artificial intelligence, and machine learning [19,20,21]. Among passive remote sensing methods, hyperspectral sensing, which records reflected solar radiation, shows great potential [22,23]. Hyperspectral sensors use tens or hundreds of narrow channels covering 400–2500 nm (VIS, NIR, SWIR) [24,25].

In recent years, hyperspectral imaging has been increasingly used for the early detection of plant diseases [26]. The technology has been successfully applied to diseases caused by micromycetes [27,28], viruses [29,30], and bacteria [31,32], as well as to assess abiotic stress [33,34].

In their previous work, the authors demonstrated the feasibility of early wheat brown rust (*Puccinia triticina*) detection using detached leaves [35] and early wheat stem rust detection using seedlings [36]. It was shown that hyperspectral preprocessing and machine learning are critical for successful early disease detection [36]. Despite the rapid progress in hyperspectral imaging for plant disease diagnostics, most existing studies are limited to single crops or specific experimental setups, which restricts their practical applicability. A critical gap remains in assessing whether diagnostic models trained on one crop can be reliably transferred to another, closely related species without loss of accuracy. Addressing this challenge is of both scientific and practical importance: it opens the way to creating universal, cost-efficient diagnostic tools applicable across multiple cereal crops. The present study is among the first to systematically evaluate cross-crop transferability of machine learning models for hyperspectral disease detection, thereby providing novel insights into the universality of spectral responses to rust infections and paving the way for scalable, field-ready monitoring systems.

Thus, the objectives of this study were: to validate the feasibility of early detection of barley stem rust using hyperspectral data processing and machine learning methods previously tested for wheat stem rust; to compare spectral characteristics of healthy and infected wheat and barley plants; to evaluate the transferability of algorithms trained on wheat datasets for diagnosing stem rust in barley and to identify patterns enabling application of the developed methods to other crops.

## 2. Materials and Methods

Wheat stem rust inoculum (infected wheat plants with urediniopustules of *P. graminis* f. sp. *tritici*) was collected in July 2024 from commercial spring bread wheat fields in the Chelyabinsk region, Russian Federation. The plants were preserved as herbarium samples for subsequent pathogen multiplication. The fungal population was propagated under laboratory conditions. The universally susceptible winter bread wheat cultivar "Michigan Amber" was used for fungal propagation. The stem rust pathogen was multiplied from herbarium material on 10-day-old intact wheat seedlings (first leaf stage) using established methods [37]. Urediniospores were collected by gently tapping them into glass tubes and used for plant inoculation. Inoculation was performed using freshly collected urediniospores. For better simulation of field conditions, a population of *P. graminis* f. sp. *tritici* was used rather than single isolates.

The study utilized spring bread wheat cv. “Saratovskaya 74” and spring barley cv. “Lyuboyar,” both susceptible to *P. graminis* f. sp. *tritici*. In each experiment, wheat and barley were sown in 12 plastic containers (320 × 220 × 160 mm) with 75 seeds per container, which corresponds to the hectare seeding rates for these varieties, based on the varieties’ description and container area. Each experiment was repeated three times. Containers were divided into two equal groups (control and inoculated). Plants were maintained under optimal conditions (watering, fertilization) in a phytotron at 23–25 °C, with a 16 h photoperiod and photosynthetic photon flux density of 202.5–270 µmol m^−2^ s^−1^ (15,000–20,000 lux), and 60–70% humidity.

Plants were inoculated with fungal urediniospores at growth stage 12 on the BBCH scale. Plants were sprayed with a spore suspension (10^4^ spores/mL) with NOVEC 7100 hydrofluoroether additive, using an airbrush. The plants were then covered with a polyethylene frame to create a humid chamber and kept in the dark at 20–23 °C for 24 h. Afterward, the polyethylene was removed, and the plants were incubated under the same phytotron conditions [38].

Visible stem rust symptoms were observed from the 6th to the 10th day after inoculation. Phytopathological assessment was performed according to standard laboratory protocols for seedling processing [39]. Typically, the infection type is recorded 10–12 days after infection. On the 10th day after infection, the infection type corresponded to a score of 4 on the standard 0 to 4 Stakman scale [40] for the “Saratovskaya 74” cultivar and 3+ on the modified barley scale [41] for the "Lyuboyar" cultivar, indicating high susceptibility to stem rust in the analyzed cultivars. The severity of stem rust infection was assessed using the modified Cobb scale after Peterson [42], ranging from 70 to 100 in all conducted experiments. 

Hyperspectral imaging was performed in a light-isolated room. The camera was mounted horizontally on a tripod 0.5 m above the samples. Samples were illuminated by two 500 W halogen lamps at 45°. A dark background was used as optimal for imaging plant material [43]. The frame area was 20 × 20 cm. The setup design followed established protocols [44,45,46].

Experiments employed the Ultris 20 hyperspectral snapshot camera (Cubert GmbH, Ulm, Germany), acquiring data in the 450–874 nm range with 106 channels at 4 nm intervals. Image resolution was 410 × 410 pixels. Calibration included black/white standards, distance calibration, and proprietary Cubert-Pilot software version 2.8.1 (Cubert GmbH, Ulm, Germany).

Images were taken daily at 12:00 from day 3 to day 8 post-inoculation. This interval was chosen as it spans the onset of reliable classification before visible symptom development. Each daily dataset (control + infected) contained 144 images. The complete dataset comprised 864 images (432 healthy, 432 diseased). Data were saved in Multi-Channel TIFF format (106 channels, 16-bit).

## 3. Results

The analysis of experimental data was carried out to address the main objectives of the study: (i) to evaluate the effect of spectral preprocessing on the quality of diagnostic features; (ii) to assess the discriminative ability of different feature spaces derived from hyperspectral signatures; (iii) to determine the classification performance of machine learning models; and (iv) to test the transferability of models between wheat and barley.

To achieve these goals, the results are presented in the following order. First, we describe the preprocessing workflow and illustrate how each step contributes to data quality improvement. Then, we compare alternative feature construction strategies and analyze their impact on classification accuracy. Finally, we evaluate the performance of different machine learning models and summarize the outcomes of cross-crop validation experiments, which are critical for assessing the scalability of the proposed approach.

### 3.1. Data Processing

#### 3.1.1. Hyperspectral Data Processing Workflow Scheme

In this study, hyperspectral image cubes with dimensions of 864 × 410 × 410 × 106 were employed for each dataset, where 410 × 410 represents the spatial resolution and 106 denotes the number of spectral bands. In total, six independent datasets were collected: three for wheat and three for barley. Each dataset consisted of 864 hyperspectral cubes, acquired between the 3rd and 8th day after pathogen inoculation.

**Figure 1:**
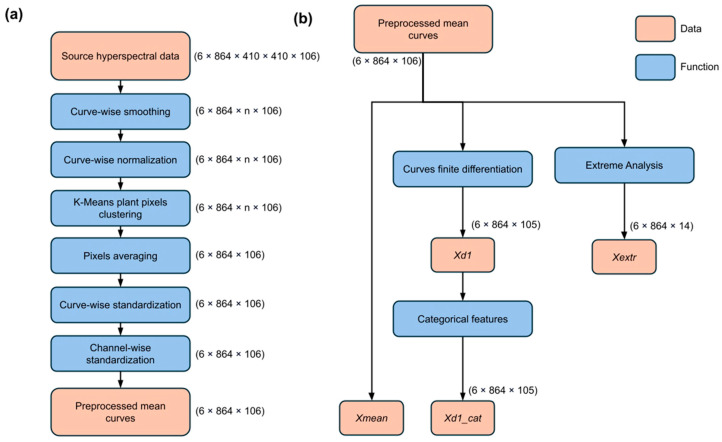
Hyperspectral data processing workflow scheme: preprocessing and feature extraction/engineering. (**a**) Complete preprocessing hyperspectral data pipeline workflow. (**b**) Feature space construction scheme.

The data processing workflow consisted of two main stages: preprocessing and feature extraction/engineering, and is presented in Figure 1.

During the preprocessing stage (Figure 1a), operations were performed to enhance data quality, including spectral normalization, noise reduction, and smoothing, which resulted in the generation of mean spectral signatures. The subsequent feature extraction stage (Figure 1b) was designed to transform these spectral signatures into informative features for machine learning, employing approaches such as dimensionality reduction and spectral feature engineering to improve discriminative power.

#### 3.1.2. Hyperspectral Images Segmentation

To isolate objects in the hyperspectral data, image segmentation was performed based on pixel clustering in the 106-dimensional spectral space. For each hyperspectral cube, the K-means algorithm was applied with a fixed number of clusters (k = 2). The resulting clusters were interpreted as follows: the background corresponded to the cluster with the lowest reflectance values, while the plant material was assigned to the cluster with the highest reflectance.

Formally, the set of pixels *Ps*, corresponding to the object in the s-th hyperspectral image *Hs*, was defined as

$$P_{s}=\left\{p\in H_{s}|k_{s}\left(p\right)=\zeta_{s}^{max}\right\}$$

where *ks* denotes the K-means clustering function; $\zeta_{s}^{max}$—s is the index of the cluster with the highest spectral variance, and p represents a spectral pixel (a vector in $R^{106}$).

#### 3.1.3. Spectral Curve Smoothing

The Savitzky–Golay filter [47] was applied to smooth spectral signatures and reduce noise impact. This method approximates the values within a sliding window of width 2 M + 1 using a polynomial of degree N, while preserving local spectral characteristics of the signal.

The smoothing procedure is based on minimizing the approximation error between the original values $f\left(x_{m}\right)$ and the polynomial $p_{N}\left(x_{m}\right)$ using the least-squares criterion:

$$E_{N}=\sum_{m=-M}^{M}{\left[p_{N}\left(x_{m}\right)-f\left(x_{m}\right)\right]}^{2}$$

where *E_N_* is the approximation error minimized through optimization of the polynomial coefficients *c_n_*. The key parameters of the filter are the window size and the polynomial order.

#### 3.1.4. Spectral Curves Standardization

To eliminate differences in scale and variance among features, the spectral curves were standardized using the Standard Normal Variate (SNV) transformation [48]. Let denote the spectral curve of image *s*, where $x_{s,c}$ is the reflectance value at channel *c*. The standardization is performed according to the following:

$$C_{s}={\left\{x_{s,c}\right\}}_{c=1}^{106}$$

$$f^{cu}\left(x_{s,c}\right)=\frac{x_{s,c}-\mu_{s}}{\sigma_{s}}$$

where $\mu_{s}$ and *σs* are the mean and standard deviation of the spectral curve *Cs*, respectively.

**Figure 2:**
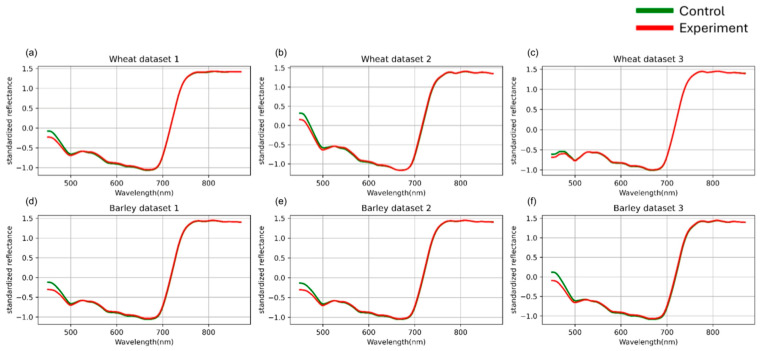
Mean spectral curves of the control and experimental groups of three wheat (**a**–**c**) and three barley (**d**–**f**) datasets averaged after SNV standardization. Control represents healthy plants, experimental represents plants inoculated with stem rust. The vertical scale shows the reflectivity expressed in normalized values, and the horizontal scale shows the spectrum wavelengths expressed in nanometers.

For each image, an average spectral curve was computed by aggregating the reflectance values of all pixels assigned to the “plant material” cluster. The average raw hyperspectral curves for each dataset are presented in Figure 2.

#### 3.1.5. Spectral Curves Channel-Wise Standardization

To equalize the variance across spectral channels while preserving their mean values, a channel-wise variance normalization procedure was applied. Let denote the set of spectral observations, where *s* is the sample index and *c* is the spectral channel index. For each channel *c*, the mean *μc* and the standard deviation *σc* were computed. The standardization was then performed according to the following:

$$X=\left\{x_{s,c}\right\},\;s=1,\ldots ,N,\;c=1,\ldots ,C$$

$$f^{ch}\left(x_{s,c}^{mean}\right)=\frac{x_{s,c}^{mean}-\mu_{c}}{\underset{c∊1...106}{\max}\sigma_{c}}$$

where $x_{s,c}^{mean}$ is the mean reflectance value of channel *c* for observation *s*, *μc* is the mean reflectance for channel *c*, and $\underset{c}{\max}\sigma_{c}$ is the maximum standard deviation across all channels.

**Figure 3:**
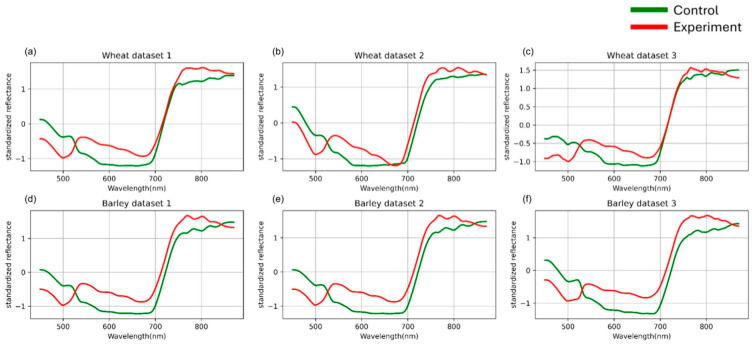
Mean spectral curves of the control and experimental groups of three wheat (**a**–**c**) and three barley (**d**–**f**) datasetsafter channel-wise standard normalization. Control represents healthy plants, and experimental represents stem rust inoculated plants. The vertical scale shows the reflectivity expressed in normalized values, and the horizontal scale shows the spectrum wavelengths expressed in nanometers.

Based on the authors’ previous findings [36], the highest classification accuracy was achieved when data were grouped by days after inoculation. Therefore, in the present study, this grouping strategy was employed for both standardization and model evaluation. The preprocessing results are presented in Figure 3.

### 3.2. Feature Construction

#### 3.2.1. First-Order Derivatives

The primary differences between classes were manifested mainly in the shape of the hyperspectral curves rather than in the absolute reflectance values. Therefore, the feature space construction was based on the spectral curve morphology analysis. To capture these shape-related variations, first-order derivatives were computed for each averaged hyperspectral curve, characterizing the local spectral dynamics.

Let *X^d^*^1^ denote the matrix consisting of N vectors of length 106, where each row corresponds to the first derivative of a curve from the set *X*mean.

$$x_{s,c}^{d1}=x_{s,\;c+1}^{mean}-x_{s,c}^{mean}$$

**Figure 4:**
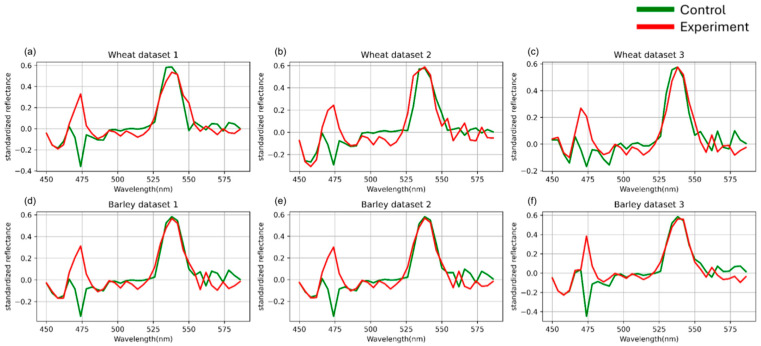
First-order derivative spectral curves of three wheat (**a**–**c**) and three barley (**d**–**f**) datasets. Control represents healthy plants, and experimental represents stem rust inoculated plants. The vertical scale shows the reflectivity expressed in normalized values, and the horizontal scale shows the spectrum wavelengths expressed in nanometers.

The resulting derivative-based features are illustrated in Figure 4.

#### 3.2.2. Categorical Derivatives

Following the computation of first-order spectral derivatives, categorical features were constructed to capture the qualitative nature of local spectral variations. Each derivative value $x_{s,c}^{d1}$ was assigned to a categorical variable $x_{s,c}^{cat}$ according to the following rules:

• 1—positive change,
• −1—negative change,
• 0—stability (value close to zero within a precision of three decimal places).

**Figure 5:**
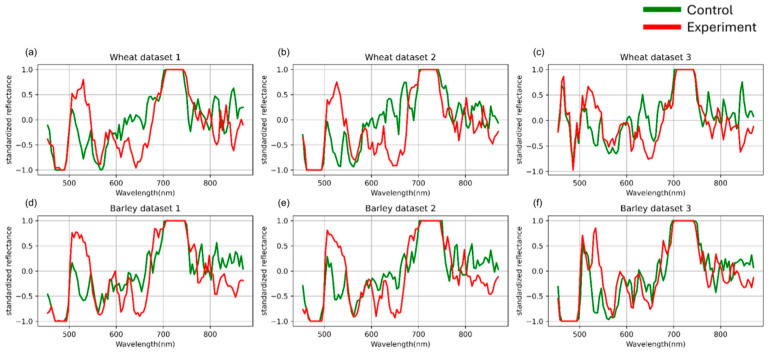
Averaged categorical features derived from the spectral curve derivatives of wheat (**a**–**c**) and three barley (**d**–**f**) datasets. Control represents healthy plants, and experimental represents stem rust-inoculated plants. The vertical scale shows the reflectivity expressed in normalized values, and the horizontal scale shows the spectrum wavelengths expressed in nanometers.

A visual representation of the transformed data is provided in Figure 5.

#### 3.2.3. Extremal Features

**Figure 6:**
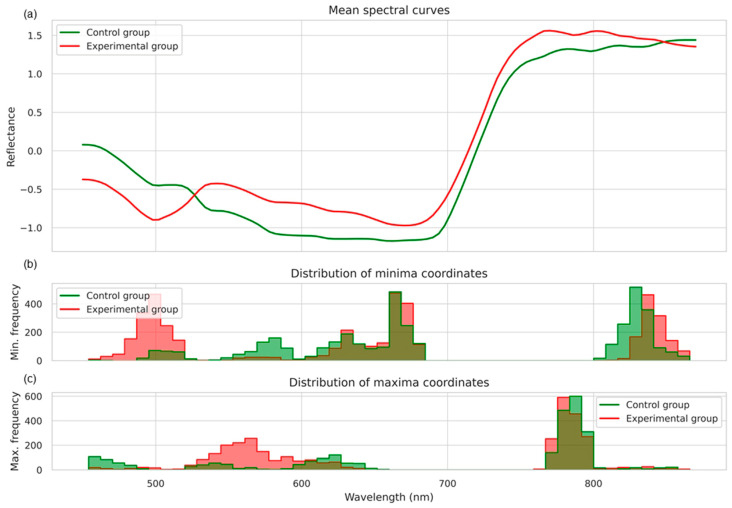
Mean spectral curves (**a**) and the distribution of local minima (**b**) and local maxima (**c**) coordinates for the control and experimental groups. Control represents healthy plants, experimental represents stem rust inoculated plants.

To construct features describing the spectral curves’ morphology, an analysis of extrema was performed on the smoothed, averaged spectra obtained for each hyperspectral image. Smoothing was carried out using the Savitzky–Golay filter, after which local minima and maxima were identified. The distribution of minima and maxima coordinates is shown in Figure 6.

Figure 6a presents the averaged reflectance spectral curves for the control and experimental groups. As shown in Figure 6b, in the experimental group, local minima were more frequently observed within the 480–520 nm and 680–720 nm ranges, whereas in the control group, minima were predominantly concentrated in the 620–650 nm and 800–830 nm regions. Figure 6c illustrates the distributions of local maxima. In both groups, the majority of maxima occurred in the near-infrared region (approximately 780–800 nm); however, the experimental group exhibited a slight shift toward shorter wavelengths and a greater spread of values.

**Figure 7:**
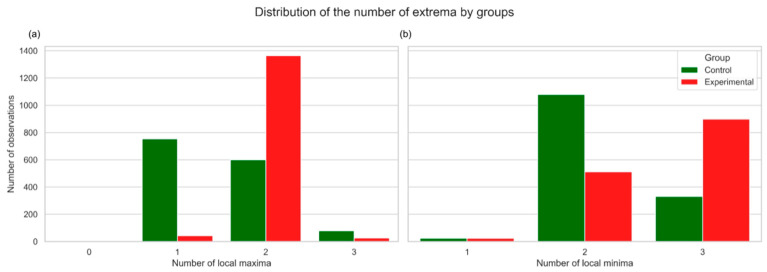
Local maxima (**a**) and local minima (**b**) number distribution, which shows the number of local extrema. Spectral curves in the experimental group are more frequently characterized by a higher complexity, with an increased number of minima and maxima. Control represents healthy plants, and experimental represents stem rust-inoculated plants.

Furthermore, as demonstrated in Figure 7, group differences were also reflected in the number of local extrema: spectral curves in the experimental group were more frequently characterized by a higher complexity, with an increased number of minima and maxima.

Based on features such as the extrema corresponding wavelengths, the number of maxima and minima, and the average distance between adjacent extrema, the objects from the control and experimental groups could be effectively distinguished. Using these informative characteristics, a new feature space describing the morphology of spectral curves was constructed, while maintaining a significantly lower dimensionality compared to the original hyperspectral data.

### 3.3. Machine Learning Models

To classify the spectral curves, the following machine learning models were employed.

The Support Vector Machine (SVM) [49] is a linear classification model based on maximizing the margin of the separating hyperplane between classes. To capture nonlinear relationships, kernel functions such as the radial basis function (RBF) and polynomial kernels were applied. The key hyperparameters considered included the kernel type and the regularization parameter.

Logistic Regression (LR) [50] is a linear classification model, where different regularization norms (L1, L2) and solver types were used as tunable hyperparameters.

The Light Gradient Boosting Machine (LGBM) [51] is a gradient boosting framework [52] optimized for computational efficiency in terms of speed and memory by incorporating Gradient-based One-Side Sampling (GOSS) and Exclusive Feature Bundling (EFB). Its hyperparameters included the number of trees, learning rate, and L1- and L2-regularization coefficients.

The model choice was motivated by their proven effectiveness in hyperspectral data classification, as demonstrated in the authors’ previous studies [35,36]. The complete hyperparameter ranges are presented in Section 3.3.2.

#### 3.3.1. Research Methodology

The training of machine learning models was carried out using three datasets of wheat spectral curves. To evaluate the generalization ability of the models, a pairwise cross-validation scheme across datasets was employed. A total of six cross-validation rounds were conducted (corresponding to all three possible dataset pairs, where in each case one dataset was used for training and the other for validation, and then the roles were reversed).

Bayesian optimization [53] was applied for hyperparameter tuning of the machine learning models. Each configuration was evaluated using the average F1-score, computed over the full cross-validation procedure. The optimal configuration was defined as the one achieving the highest mean F1-score.

**Figure 8:**
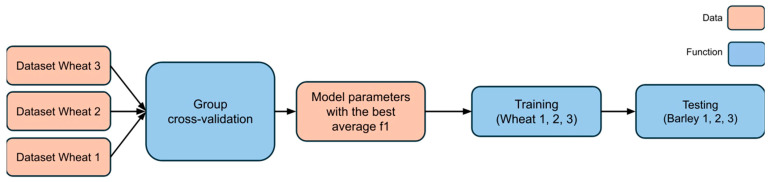
Model hyperparameter optimization scheme using group-wise cross-validation on wheat datasets followed by independent testing on barley datasets.

The model with the optimized hyperparameters (Figure 8), obtained from cross-validation on the wheat datasets, was subsequently tested on two independent barley datasets. This allowed for an objective assessment of the model’s ability to generalize to a new type of input data.

#### 3.3.2. Hyperparameter Optimization

Bayesian optimization is a global optimization approach that constructs a probabilistic surrogate model of the objective function. New parameter configurations are selected according to an acquisition (utility) function, which provides an effective balance between exploring the search space and exploiting the accumulated knowledge of model behavior. The hyperparameter search ranges used for tuning each of the considered models are summarized in Table 1.

#### 3.3.3. Model Evaluation

To assess the performance of the machine learning models, the following metrics were employed: Recall, Precision, and the F1-score.

Recall measures the proportion of correctly identified positive samples:

$$Recall=\frac{TP}{TP+FN}$$

where *TP* is the number of true positives and *FN* is the number of false negatives.

Precision indicates the proportion of predicted positive samples that are truly positive:

$$Precision=\frac{TP}{TP+FP}$$

where *FP* is the number of false positives.

The F1-score, used as the primary evaluation metric in this study, represents the harmonic mean of Precision and Recall:

$$F1=2\cdot \frac{Precision\cdot Recall}{Precision+Recall}$$

In addition, a confusion matrix (Table 2) was utilized to analyze model performance in greater detail. The matrix illustrates the distribution of correct and incorrect classifications across classes, providing insights into the relative prevalence of false negatives and false positives.

### 3.4. Classification Results Evaluation

A statistical analysis was conducted to evaluate the contribution of preprocessing steps to the classification performance across different feature spaces. Four feature spaces derived from spectral characteristics were compared, along with multiple machine learning algorithms, including Support Vector Machines (SVM), Logistic Regression (LR), and Light Gradient Boosting Machine (LGBM). The detailed procedures for feature construction and model parameterization are described in the Materials and Methods section.

#### 3.4.1. Sequential Preprocessing Steps Contribution to Model Performance

The sequence of preprocessing stages was determined by the nature of variability sources in the data. The process began with pixel-wise standardization, which mitigated local illumination variations and image noise. At the next stage, standardization of averaged curves was performed, normalizing individual differences between samples without altering the overall spectral shape. The final stage, channel-wise standardization, was applied to the already averaged and stabilized spectra, ensuring comparability across wavelengths and enabling the model to focus on relative differences between spectral ranges.

**Figure 9:**
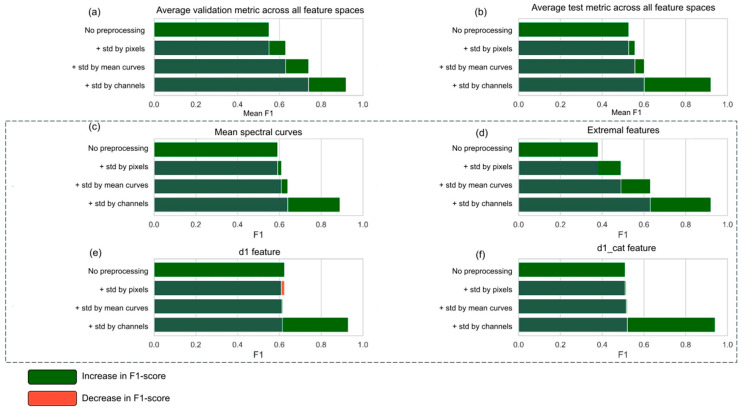
The dependence of classification accuracy on spectral data preprocessing methods. (**a**) shows F1-score on the validation set (wheat), averaged across all feature spaces for each combination of preprocessing stages. (**b**) shows F1-score on the test set (barley), averaged across all feature spaces. (**c**–**f**) show mean F1-scores on the test set (barley), calculated separately for each feature space: averaged curves (**c**), extremal features (**d**), first-order derivatives (**e**), and categorical derivatives (**f**). For each group, the dynamics of classification performance are shown as additional preprocessing stages were applied: pixel standardization, curve standardization, and channel-wise standardization. Horizontal scale represents F1 scores.

For all feature spaces, the effect of the preprocessing stages on the F1-score was evaluated (Figure 9).

The analysis demonstrated that the sequential addition of preprocessing steps—including pixel standardization, curve standardization, and channel-wise standardization—generally led to an increase in the F1-score. The highest values were achieved when the full preprocessing pipeline was applied.

The impact of preprocessing varied depending on the structure of the feature space. For mean spectral curves and extremal features, the incremental addition of preprocessing steps resulted in a consistent improvement of F1, with peak values of 0.897 and 0.926, respectively, obtained under full preprocessing. For first-order derivatives, a decline in F1 was observed at intermediate stages; however, channel-wise standardization produced a substantial gain, yielding a final score of 0.929. In the case of categorical first-order derivatives, the F1-score remained near 0.5 until the final stage, after which a sharp improvement was observed (0.94).

Across all feature spaces, the most significant contribution to performance improvement was consistently provided by channel-wise standardization.

#### 3.4.2. Comparison Across Feature Spaces

**Figure 10:**
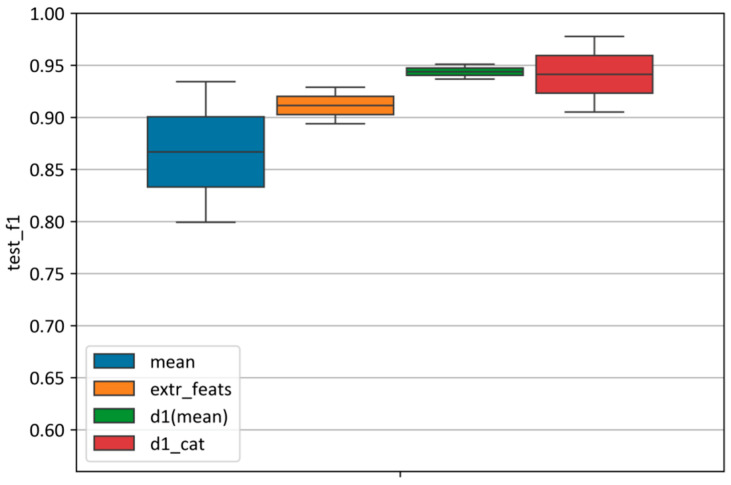
Distribution of the F1-score across different feature spaces. For each feature space, the distribution of F1-scores obtained on the test set is shown, based on the best model parameters selected according to the validation metric. “Mean” represents mean spectral curves; “extr_feats” represents extremal features; “d1(mean)” represents the first-order derivatives, and “d_cat” represents the categorical first-order derivatives.

Figure 10 illustrates the differences in classification performance among the four feature spaces. Features derived from mean spectral curves achieved an F1-score of 0.87 on the test set, though with the widest variability (ranging from 0.80 to 0.94). In contrast, extremal features provided a more stable performance (F1 ≈ 0.91), with a narrower range (0.90–0.93). The first-order derivatives yielded the most stable and highest classification accuracy (F1 = 0.94), with a very tight range (0.94–0.95). The categorical first-order derivatives also reached a high score (F1 = 0.94), but with greater variability (0.91–0.98).

#### 3.4.3. Comparison Across Machine Learning Models

**Figure 11:**
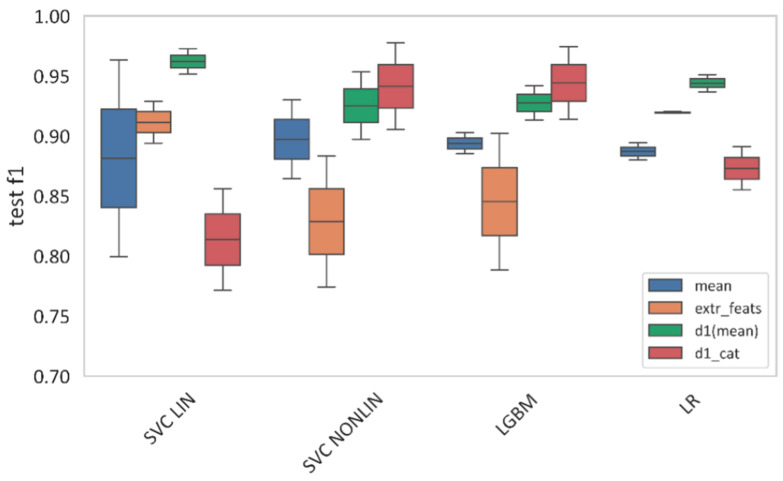
F1-score comparison across machine learning models. “Mean” represents mean spectral curves; “extr_feats” represents extremal features; “d1(mean)” represents the first-order derivatives, and “d_cat” represents the categorical first-order derivatives. “SVC LIN” and “SVC NONLIN” correspond to the best linear and nonlinear SVM models, respectively. “LGBM” and “LR” correspond to the best Light-Gradient boosting and Logistic Regression models, respectively.

Figure 11 illustrates the dependence of machine learning model performance on the type of feature space. The best results varied depending on the feature representation and the chosen model.

For mean spectral features, the highest F1-score (0.897) was achieved with the nonlinear Support Vector Machine (SVM). Gradient Boosting (LGBM) reached 0.894, Logistic Regression yielded 0.887, while the linear SVM performed slightly worse than its nonlinear counterpart (0.881 vs. 0.897).

In the extremal feature space, the best performance (F1 = 0.920) was obtained with Logistic Regression. The linear SVM achieved 0.911, LGBM 0.845, and the nonlinear SVM 0.829.

For first-order derivatives, the linear SVM achieved the best result (F1 = 0.962). Other models showed the following values: nonlinear SVM—0.925, LGBM—0.927, and Logistic Regression—0.944.

In the case of categorical first-order derivatives, the nonlinear SVM reached F1 = 0.941. The other models performed as follows: LGBM—0.944, linear SVM—0.814, and Logistic Regression—0.873.

The Gradient Boosting method (LGBM) demonstrated stable performance across all feature spaces, with F1-scores ranging from 0.845 to 0.944.

The analysis revealed the following patterns: Linear methods (Logistic Regression and linear SVM) are effective for extremal features and first-order derivatives. Nonlinear methods (nonlinear SVM) outperform linear models on mean spectral features and categorical derivatives. LGBM provides consistently robust performance across all feature spaces.

#### 3.4.4. Confusion Matrices-Based Model Generalization Analysis

To evaluate the ability of the model to generalize disease-related spectral features across different crops, a series of cross-crop experiments was conducted. In each case, the model was trained on the data of one crop (wheat or barley) and subsequently tested on the other.

Table 3 presents the results of testing on barley after training on wheat. The proportion of false positives (healthy samples classified as infected) was 5.9%, whereas the proportion of false negatives (infected samples classified as healthy) was only 1.4%.

In the second scenario (Table 4), when trained on barley and tested on wheat, the model demonstrated even higher sensitivity: only 1.9% of infected samples were misclassified as healthy. In both cases, the model made only a minimal number of errors in classifying infected plants. These results confirm that the model relies on disease-related spectral patterns rather than crop-specific characteristics, making it applicable in a cross-varietal diagnostic setting.

#### 3.4.5. Overall Outcomes

The study showed that hyperspectral imaging analysis of wheat “Saratovskaya 74” and barley “Lyuboyar” varieties inoculated with urediniospores of *Puccinia graminis* f. sp. *tritici* enabled detection of the disease on the 3rd day after inoculation, before visible symptoms appeared. The results demonstrated that the similarity of *P. graminis* pathogenesis in wheat and barley ensures the universality of spectral patterns of the disease, which allows the same models to be applied across different crops. Models trained on wheat data showed high accuracy when tested on barley (F1 > 0.94) and vice versa, with false negative classifications of less than 2%.

The data were processed using multi-stage preprocessing and spectral feature extraction, followed by classification with machine learning methods (SVM, LR, LGBM). Data preprocessing proved critical for improving classifier performance. Standardization across spectral channels made the greatest contribution to enhancing diagnostic accuracy. The complete processing chain (pixel standardization → curve averaging → channel standardization) yielded the highest F1 values. Among the feature spaces, the first-order derivatives of spectral curves achieved the best performance (F1 up to 0.962), followed by categorical derivatives (F1 up to 0.94) and extreme-value features (F1 ≈ 0.92). Among classifiers, linear SVM was optimal for first-order derivatives, logistic regression for extreme features, and nonlinear SVM for averaged spectra and categorical derivatives. LGBM provided stable but not maximal performance in all cases.

## 4. Discussion

The causal agent of stem rust, *Puccinia graminis* f. sp. *tritici*, is an obligate biotrophic fungus with a life cycle including five distinct stages of sporulation and an alternation of hosts [12]. Infection in wheat and barley follows a similar pattern: urediniospores that land on the leaf surface under high humidity conditions germinate and penetrate through stomata, after which the mycelium spreads in the intercellular space of the mesophyll, forming haustoria inside the cells to obtain nutrients [12,13]. In our experiments, we used the same population of *P. graminis* f. sp. *tritici* on both wheat and barley.

Although the general stages of stem rust pathogenesis are the same for wheat and barley, the morphology of uredinia and tissue reactions show species-specific features. On susceptible wheat varieties, pustules are usually large and elongated with abundant spore release, while in barley, they are more often oval and may be accompanied by pronounced chlorosis. The types of responses to fungal infection in barley are less clearly expressed. In addition, different types of pustules are often found on a single barley leaf, indicating a heterogeneous tissue response to infection [54].

From a physiological perspective, stem rust infection causes similar changes in both crops: a decrease in chlorophyll content, a shift in pigment ratios, disruption of water balance, and an increase in the content of free amino acids and soluble sugars [55,56,57]. These processes have a complex effect on spectral response: in the visible range (VIS), reflectance decreases in the green and red zones due to chlorophyll degradation, while in the near-infrared (NIR), reflectance decreases due to the destruction of cellular structures and loss of turgor [23,58]. Thus, biochemical and structural changes during the development of stem rust are largely universal for both crops, which explains the applicability of the same spectral features for diagnostics. Despite the high universality of spectral features, morphological and physiological differences between crops can affect diagnostic accuracy. Barley leaf blades are generally wider and shorter than wheat leaf blades and have a slightly different angle to the stem, which can influence near-infrared reflectance. In addition, differences in cuticle structure and epidermal density can alter reflectance in certain ranges.

It is important to note that the sequence of hyperspectral data preprocessing was one of the key factors determining the efficiency of stem rust classification on wheat and barley. The experimental results show that the use of individual preprocessing procedures in isolation has a smaller effect than their strictly defined combination. This finding is consistent with previous studies in phytopathology [58,59,60], which emphasize that each preprocessing stage eliminates a specific source of data variability, and that the correct order minimizes error accumulation.

The first step in our study was pixel standardization, which smoothed out local variations in illumination and suppressed noise arising from both light source properties and small defects on the leaf surface. Similar approaches are used, for example, in near-infrared spectroscopy for grain quality assessment, where pixel normalization prevents distortions caused by uneven illumination [61]. The next step, standardization of the averaged curves, eliminated individual differences between samples not related to the biological state of the plants. In particular, it leveled the influence of morphological features such as leaf thickness or its angle relative to the light source. This is especially important for intercultural comparisons, where the physical structure of wheat and barley leaves differs in thickness, cuticle density, and vein orientation [62]. The final step, standardization across bands, ensured comparability of values across spectral ranges and allowed machine learning algorithms to focus on relative changes in reflectance rather than absolute signal levels. Such procedures are standard in hyperspectral data analysis for medical diagnostics [63] and have also proven effective in agronomic applications [64]. Thus, the order of preprocessing is important because each procedure eliminates a specific class of artifacts, and their correct sequence minimizes mutual influence and error accumulation. However, for some feature spaces (e.g., categorical derivatives), strict adherence to the sequence may be less critical, since the features themselves are resistant to large-scale fluctuations.

From a mathematical perspective, the use of first-order derivatives of spectral curves allows a focus on the shape of the curve—that is, on relative changes in reflectance between adjacent wavelengths—rather than absolute values. This technique has long been used in spectroscopy to compensate for variations associated with sample thickness, light scattering, and illumination differences [48]. It removes the constant component (offset) and suppresses slowly varying components of the spectrum, which is particularly important when working with data obtained under different lighting conditions. The categorical derivatives used in our study translate derivative values into a simplified representation (increase, decrease, or stability of the signal). This reduces the dimensionality of the feature space and sensitivity to small noise while preserving key information about curve shape. Similar approaches have been successfully applied to classify plant diseases based on spectral data [33,65]. Extreme-value features (curve minima and maxima) complement derivatives by recording key points in the spectrum where reflectance changes most strongly. Biologically, these points may correspond to pigment absorption ranges (e.g., 680–720 nm for chlorophyll-a) or water absorption bands in the NIR range. Together, such features form a compact and informative spectral representation, resistant to external artifacts and suitable for cross-crop diagnostics.

One of the most important results of our study was the confirmation of the high transferability of models trained on wheat data for diagnosing stem rust on barley, and vice versa. During testing, the model trained on wheat showed high accuracy on barley (F1 > 0.94), with a minimal number of false negative classifications. This indicates that the model identified universal patterns of spectral response to infection that are independent of species-specific morphological features. A similar effect has been reported in previous studies. For example, spectral features reflecting pigment degradation and tissue structural damage during infection were shown to be similar across plant species [66]. Other researchers have noted the possibility of transferring models between wheat varieties without significant loss of accuracy [67]. In another study, a classification model developed for diagnosing citrus greening in the “Satsuma” variety was successfully applied to the “Ponkan” variety by transferring a calibration model with a low level of false negatives [31]. The practical value of high transferability lies in the possibility of significantly reducing the cost of training models for new crops or varieties. A training set from one crop may be sufficient to apply the model to another, especially for closely related species with similar physiological responses. The results obtained highlight the potential for scaling the developed approach to other crops and diseases.

Although this study did not employ explicit transfer learning or domain adaptation techniques, the cross-crop validation procedure represents a form of zero-shot domain transfer, where the model trained on one crop (source domain) was directly applied to another (target domain). This approach evaluates the generalization potential of hyperspectral features across related species rather than model fine-tuning. Thus, “cross-crop transferability” in this context refers to model generalization across domains rather than classical transfer learning.

A limitation of this study is that the experiments were conducted under controlled laboratory conditions. In the field, additional factors such as variable sunlight, background vegetation, and leaf surface contamination may reduce classification accuracy. Reliable operation in real conditions requires adaptation of models to variable imaging conditions or the use of compensation methods [68]. Another risk factor is the presence of mixed infections or simultaneous exposure to abiotic stresses (e.g., drought, nutrient deficiency), which can cause spectral changes similar to rust symptoms [69]. To increase diagnostic specificity in such cases, it is advisable to use additional features or combine hyperspectral data with other sensor types, which we plan to address in future studies.

Future research should also consider the following directions: (1) the use of sensors operating in the SWIR range (1100–2500 nm) to record absorption bands associated with water, lignin, cellulose, and other components, thereby improving diagnostic accuracy [70]; (2) field tests to evaluate the developed models under natural light conditions and in the presence of background vegetation, which is necessary to confirm practical applicability; (3) integration of hyperspectral cameras with unmanned aerial vehicles and the use of real-time algorithms to automate the rapid detection of disease foci over large areas; and (4) the study of combined features that integrate derivatives, extreme characteristics, and statistical indicators of curves to further enhance model stability and transferability.

## 5. Conclusions

This study analyzed the effects of different stages of spectral data preprocessing and constructed additional feature spaces reflecting both the shape and key features of spectral curves. Early detection of stem rust on barley was successfully achieved using hyperspectral data processing and statistical algorithms previously validated on wheat. A comparative analysis of the spectral characteristics of healthy and infected plants confirmed that consistent preprocessing, particularly channel standardization, significantly improves classification accuracy.

Different feature types responded differently to normalization stages, but in all cases, performance improved, with derivative and extreme-value features providing the highest F1 scores. The constructed feature spaces substantially reduced data dimensionality without loss of biological relevance. Importantly, models trained on wheat data retained high accuracy when applied to barley, demonstrating that the extracted features capture universal spectral signatures of stem rust independent of crop species.

The developed approach is applicable for cross-crop diagnostics of rust diseases. Optimization of feature engineering and preprocessing enhances both the accuracy and robustness of classification, offering potential for scalable and transferable tools in agricultural disease monitoring.

## Figures and Tables

**Table 1:** Hyperparameters and their search ranges for model optimization.

Model	Hyperparameter	Search Range
Logistic Regression (LR)	Penalty (regularization type)	L1, L2
	C (regularization coefficient)	[10^−3^, 10^3^]
	Solver (optimization algorithm)	liblinear, saga
Support Vector Machine (SVM)	C (regularization parameter)	[10^−3^, 10^3^]
	Kernel (Kernel type)	Linear, rbf, poly
	max_iter (number of iterations)	[10^2^, 10^5^]
Light Gradient Boosting Machine (LGBM)	n_estimators (number of trees)	[1, 700]
	learning_rate (learning rate)	[10^−5^, 10^0^]
	reg_alpha (L1-regularization coefficient)	[0, 10^3^]
	reg_lambda (L2-regularization coefficient)	[0, 10^3^]

**Table 2:** Confusion matrix structure.

	Predicted: Control	Predicted: Experimental
Actual: Control	TP (True Positives)	FN (False Negatives)
Actual: Experimental	FP (False Positives)	TN (True Negatives)

**Table 3:** Summary confusion matrix (normalized): cross-crop transfer (training on wheat, testing on barley).

	Predicted: Control	Predicted: Experimental
Actual: Control	0.941	0.059
Actual: Experimental	0.014	0.986

**Table 4:** Summary confusion matrix (normalized): cross-crop transfer (training on barley, testing on wheat).

	Predicted: Control	Predicted: Experimental
Actual: Control	0.892	0.108
Actual: Experimental	0.019	0.981

## Data Availability

Data is available via online download link https://drive.google.com/drive/folders/1qAgWEAH5BruTNmT0bmSm4HTbXe_iitap (accessed on 21 October 2025).

## Abbreviations

The following abbreviations are used in this manuscript:

BBCH	Biologische Bundesanstalt, Bundessortenamt und CHemische Industrie
EFB	Exclusive Feature Bundling
FN	False Negative
FP	False Positive
GOSS	Gradient-based One-Side Sampling
LGBM	Light Gradient Boosting Machine
LR	Logistic Regression
NIR	Near-Infrared
NOVEC	3M™ Novec™ Engineered Fluid
RBF	Radial Basis Function
SNV	Standard Normal Variate
SVM	Support Vector Machine
SWIR	Short-Wave Infrared
TIFF	Tagged Image File Format
TP	True Positive
TTKS	Ug99 lineage race of Puccinia graminis
VIS	Visible Spectrum
